# Supplementary material for: Valproic Acid Treatment after Traumatic Brain Injury in Mice Alleviates Neuronal Death and Inflammation in Association with Increased Plasma Lysophosphatidylcholines
Source: Cells. 2024 Apr 23;13(9):734. doi: 10.3390/cells13090734 (PMC11083124; doi:10.3390/cells13090734)

Figure 1A

Original image

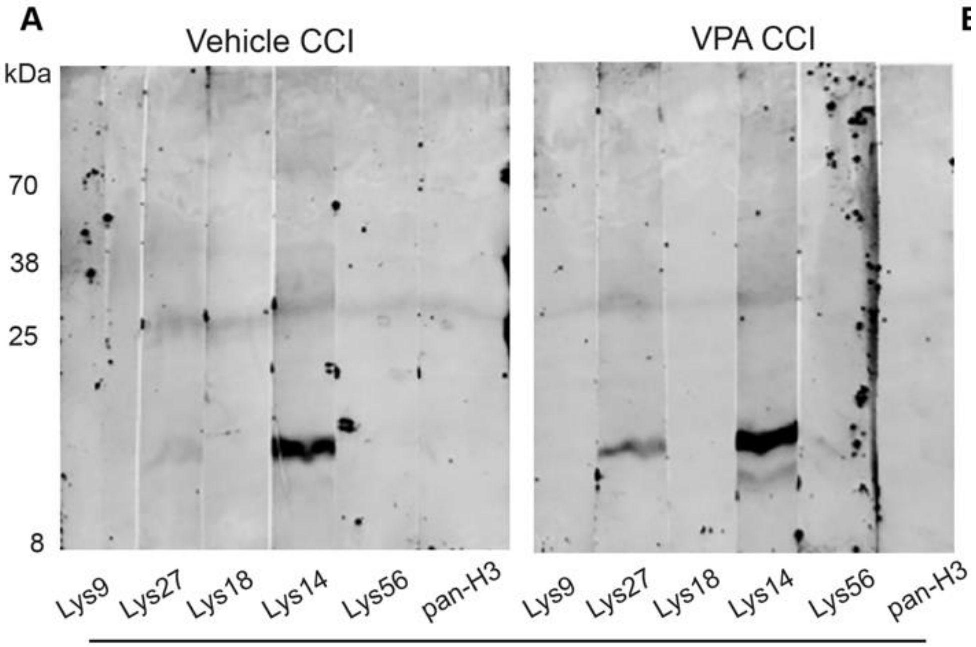

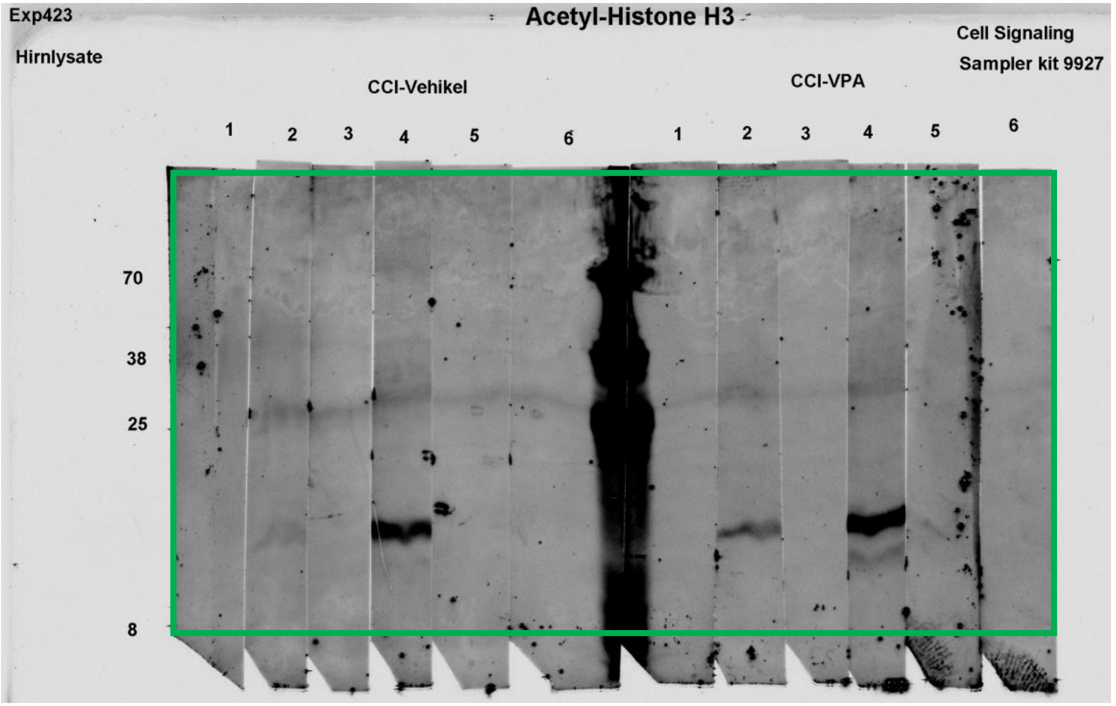

Figure 4A

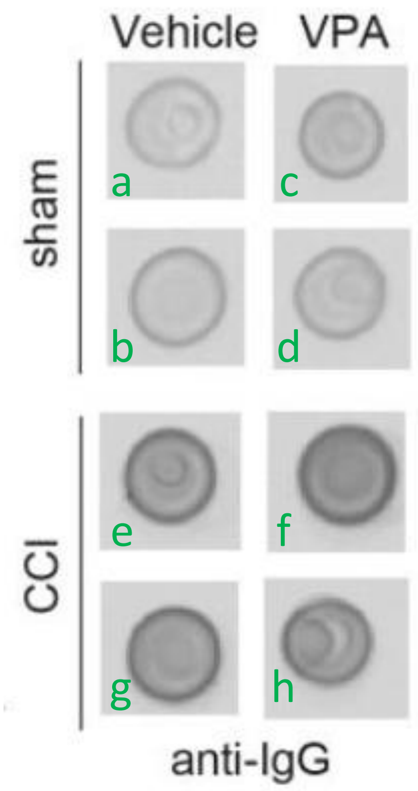

Original image

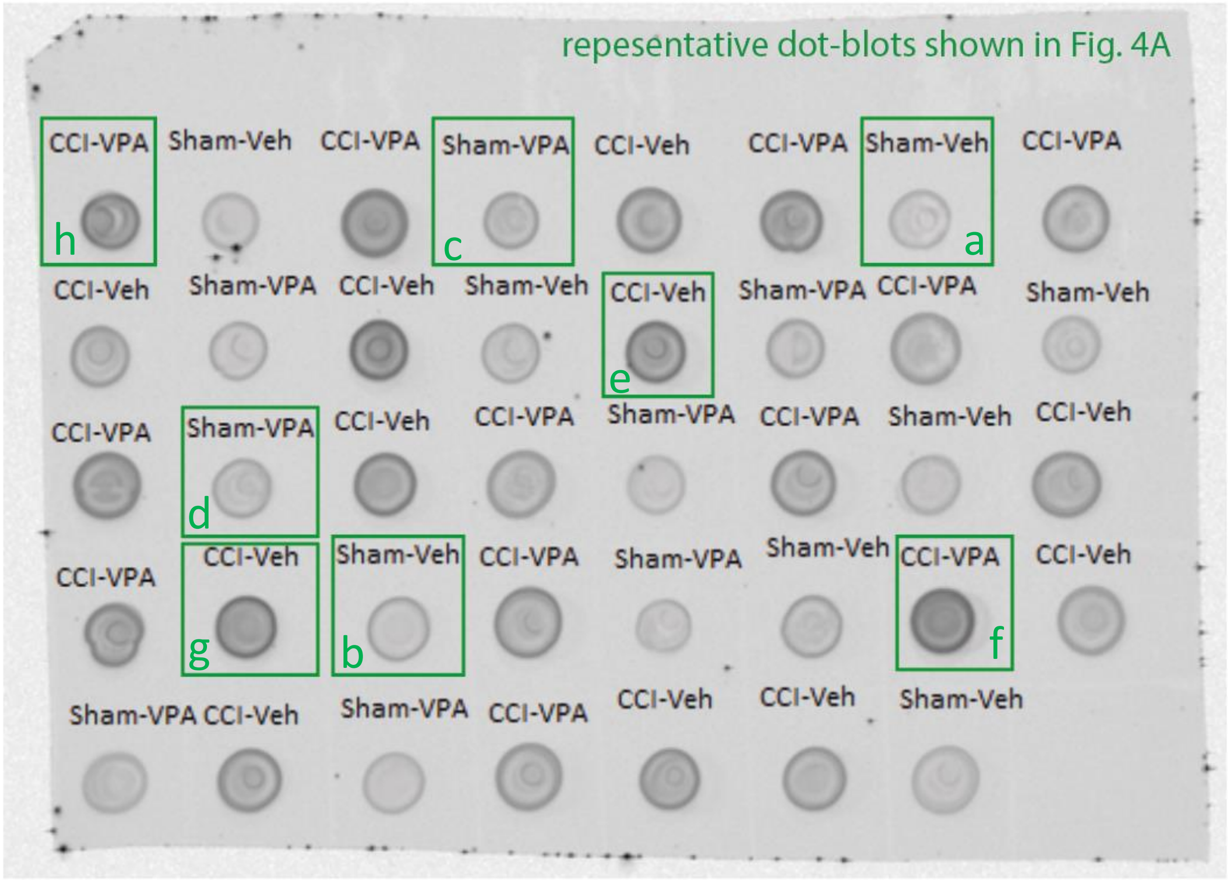

Figure 4C

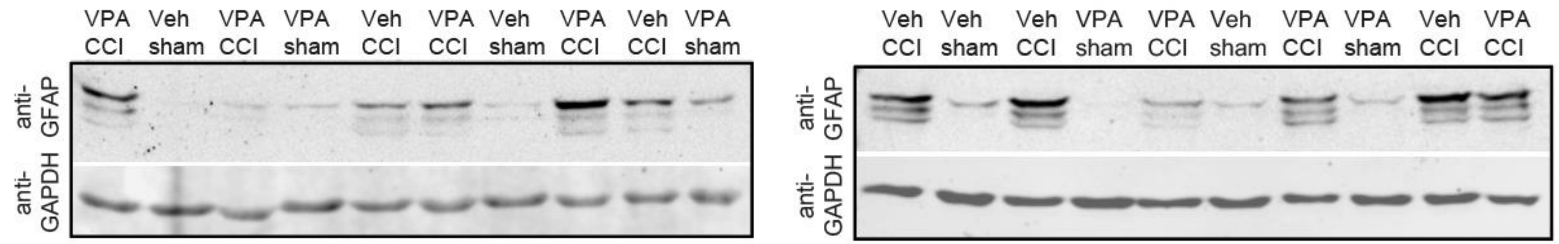

Original images

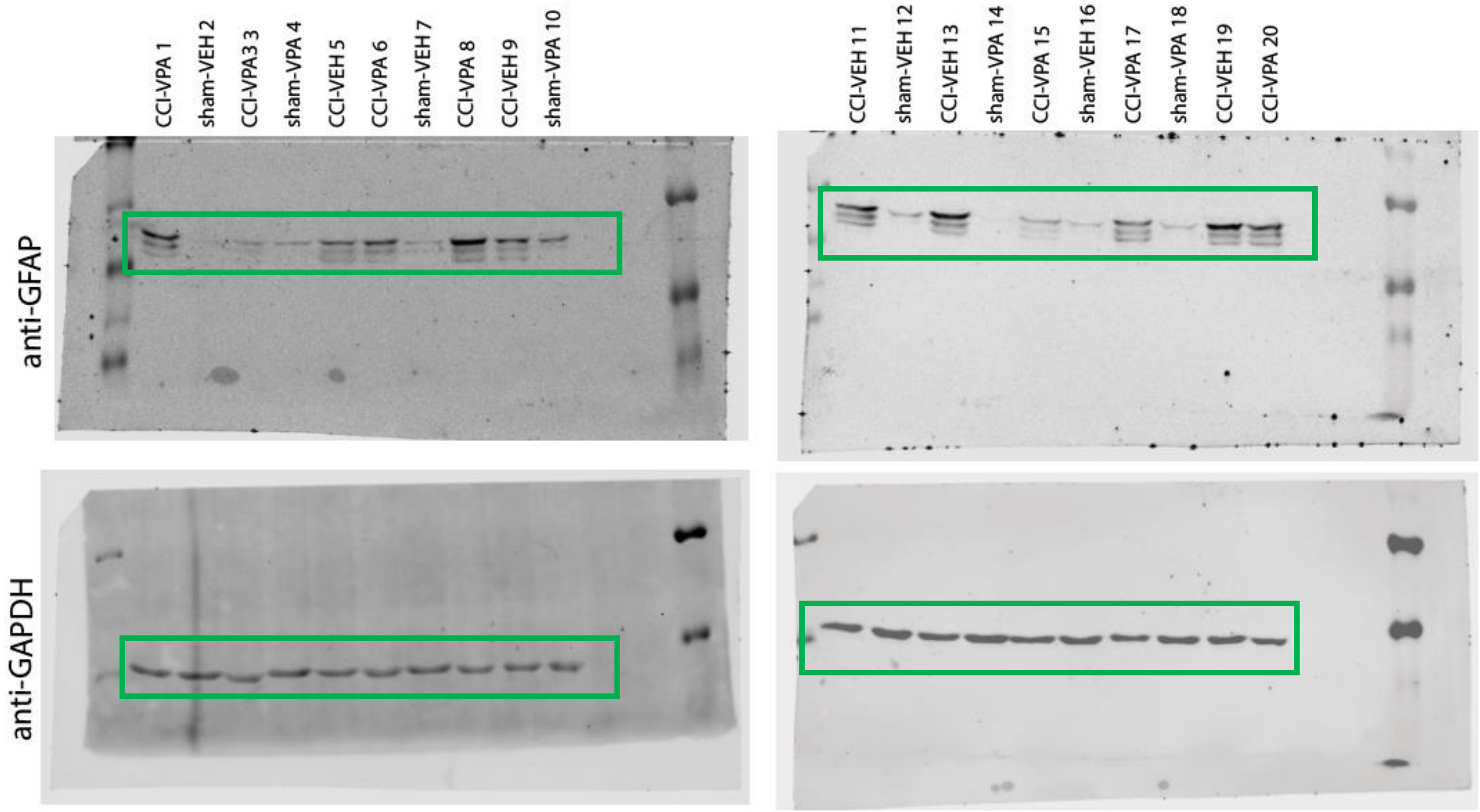

Figure 4C

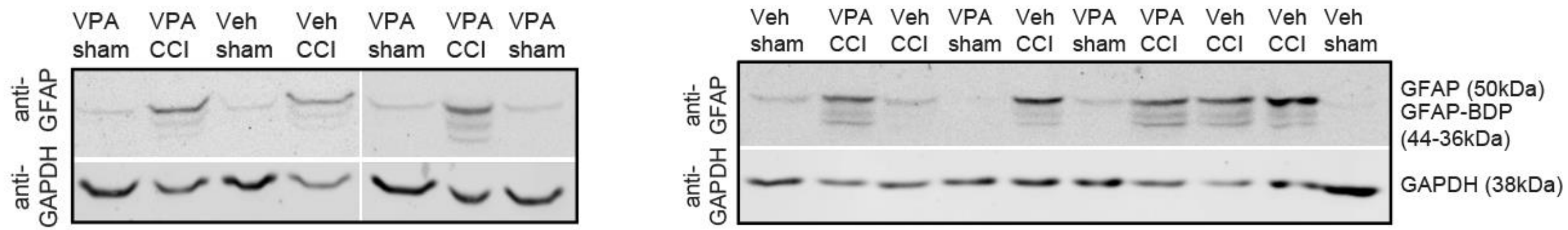

Original images

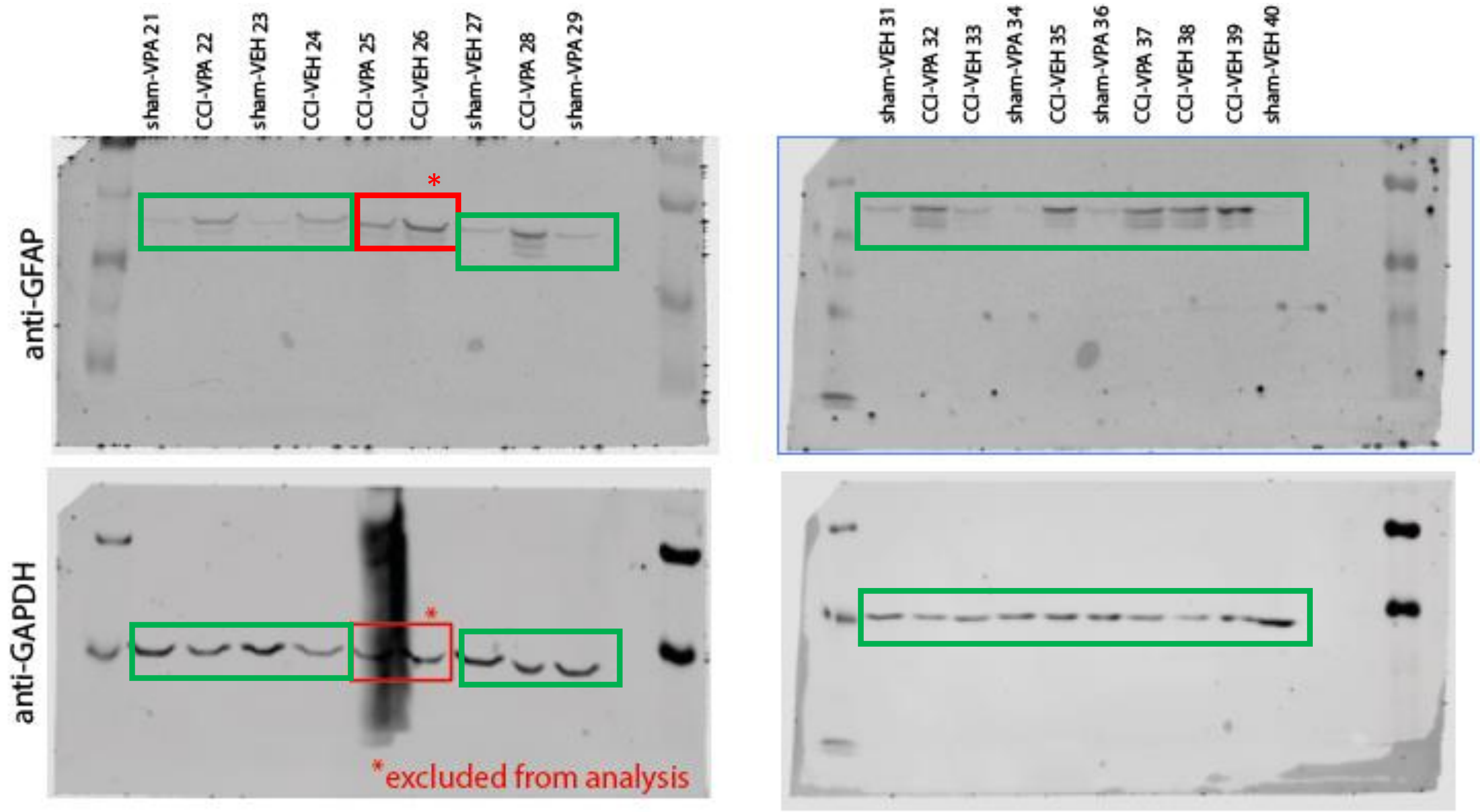

Supplement: Supplementary file 1 [file cells-13-00734-s001.zip › original and representative images.pdf]
