# Supplementary material for: Valproic Acid Treatment after Traumatic Brain Injury in Mice Alleviates Neuronal Death and Inflammation in Association with Increased Plasma Lysophosphatidylcholines
Source: Cells. 2024 Apr 23;13(9):734. doi: 10.3390/cells13090734 (PMC11083124; doi:10.3390/cells13090734)
Supplement: Supplementary file 1 [file cells-13-00734-s001.zip › Supplement-to-Hummel-et-al_R1.pdf]

Supplementary Material to

Title:

Valproic acid treatment after traumatic brain injury in mice alleviates neuronal death and inflammation in association with increased plasma lysophosphatidylcholines

Authors:

Regina Hummel[1], Erika Dorochow[2], Sonja Zander[1], Katharina Ritter[1], Lisa Hahnefeld[2,5,6], Robert Gurke[2,5,6], Irmgard Tegeder[2] *, Michael K. E. Schäfer[1, 3, 4] *

Supplementary Tables

Supplementary table S1

Table S1: Comparison of the physiological variables between VPA and vehicle groups

	Vehicle sham (0.9% NaCl)	VPA i.p. sham (400 mg/ kg)	Vehicle CCI (0.9% NaCl)	VPA i.p. CCI (400 mg/ kg)
weight preoperative [g]	23.75 ± 0.3	24.56 ± 0.2	23.87 ± 0.2	23.72 ± 0.3
rectal temperature preoperative [°C]	34.4 ± 0.2	32.94 ± 0.1	37.61 ± 0.1	37.62 ± 0.1
rectal temperature intraoperative [°C]	34.89 ± 0.2	34.56 ± 0.1	37.16 ± 0.1	37.03 ± 0.1
duration of operation [min]	15.38 ± 0.4	15.0 ± 0.0	17.73 ± 1.2	17.92 ± 0.7

VPA: valproic acid, CCI: controlled cortical impact, i.p.: intraperitoneal, values are mean ± SEM

Supplementary figures and legends

Supplementary figure S1

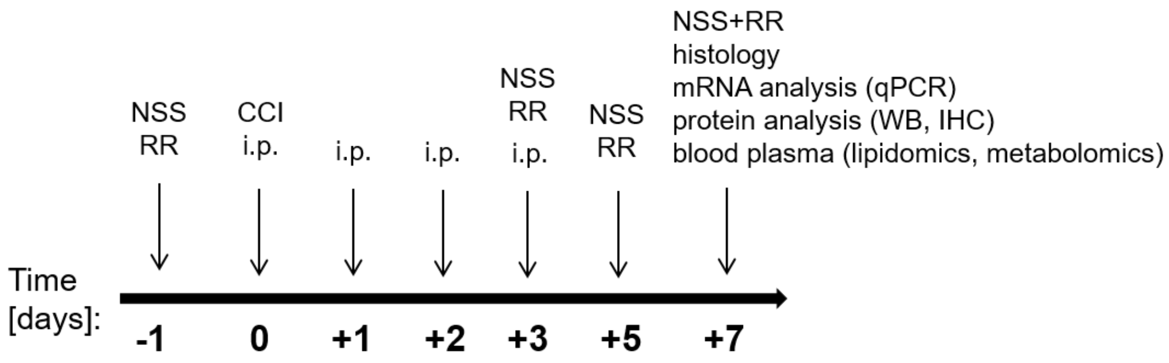

Timeline of the experiments. NSS and RR was examined at 1 day before CCI, at 1, 3, 5 and 7 days post injury (dpi). Animals received intraperitoneal (i.p.) vehicle or 400 mg/ kg body weight VPA directly after craniotomy and CCI (or sham procedure) and at 1, 2 and 3 dpi. After a survival time of 7 days, blood plasma and brains were processed for histology, mRNA, protein, lipidomic and metabolomic analyses.

Supplementary figure S2

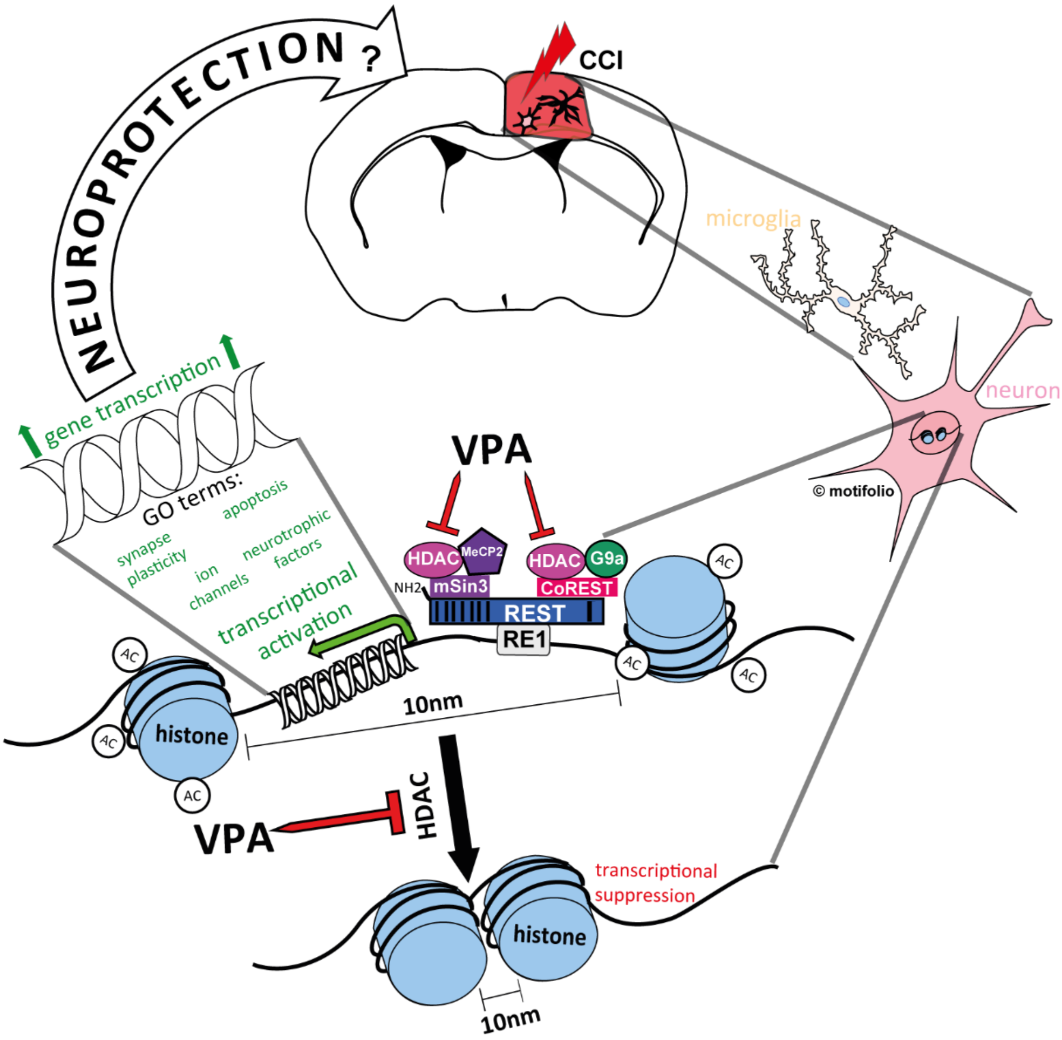

Hypothetical consequences of VPA`s HDACi function after experimental TBI. Histone acetylation and opening of the chromatin structure with transcriptional activation results in the expression of beneficial target genes, normally suppressed by the REST/ NRSF transcription factor complex (mSin3 and CoREST with further co-factors histone deacetylase (HDAC), G9a and MeCP2). Those genes may be able to generate a neuroprotective environment after a traumatic brain injury (CCI).

Supplementary figure S3

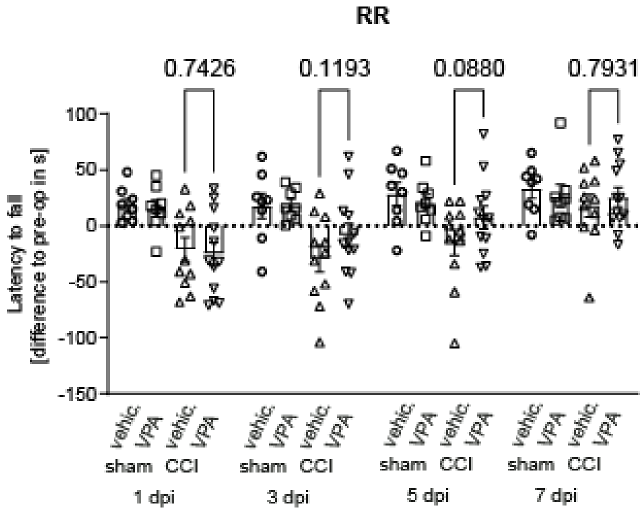

A: VPA treated animals show no difference in motor coordination after CCI. Motor coordination was analyzed by rotarod (RR) test. The differences of time spent on the accelerating rod compared with pre-operative baseline values were analyzed at 1, 3, 5 and 7 days post injury (dpi). 2-way ANOVA with post-hoc Holm-Šidák test. Sample size: vehicle CCI n=11, VPA CCI n=12, vehicle sham n=8, VPA sham n=8. Testing was carried out as described previously (Sebastiani et al., 2015).

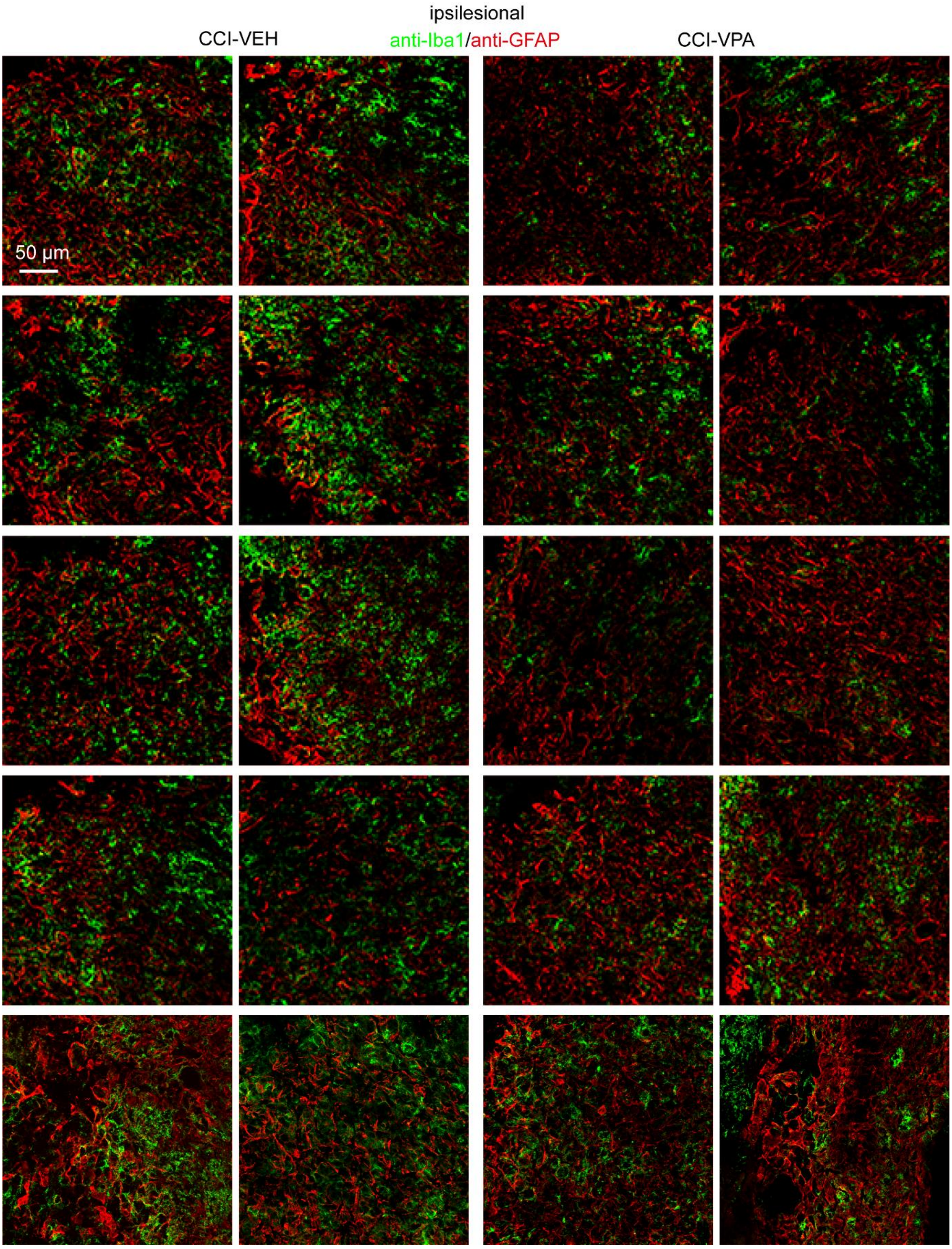

B: Exemplary panel of anti-GFAP and anti-Iba1 immunofluorescence images of the perilesional cortex in the ipsilesional hemisphere after Controlled Cortical Impact (CCI) in mice treated with vehicle or valproic acid (VPA) at 7 dpi. Images from individual mice are shown.

Supplementary figure S4

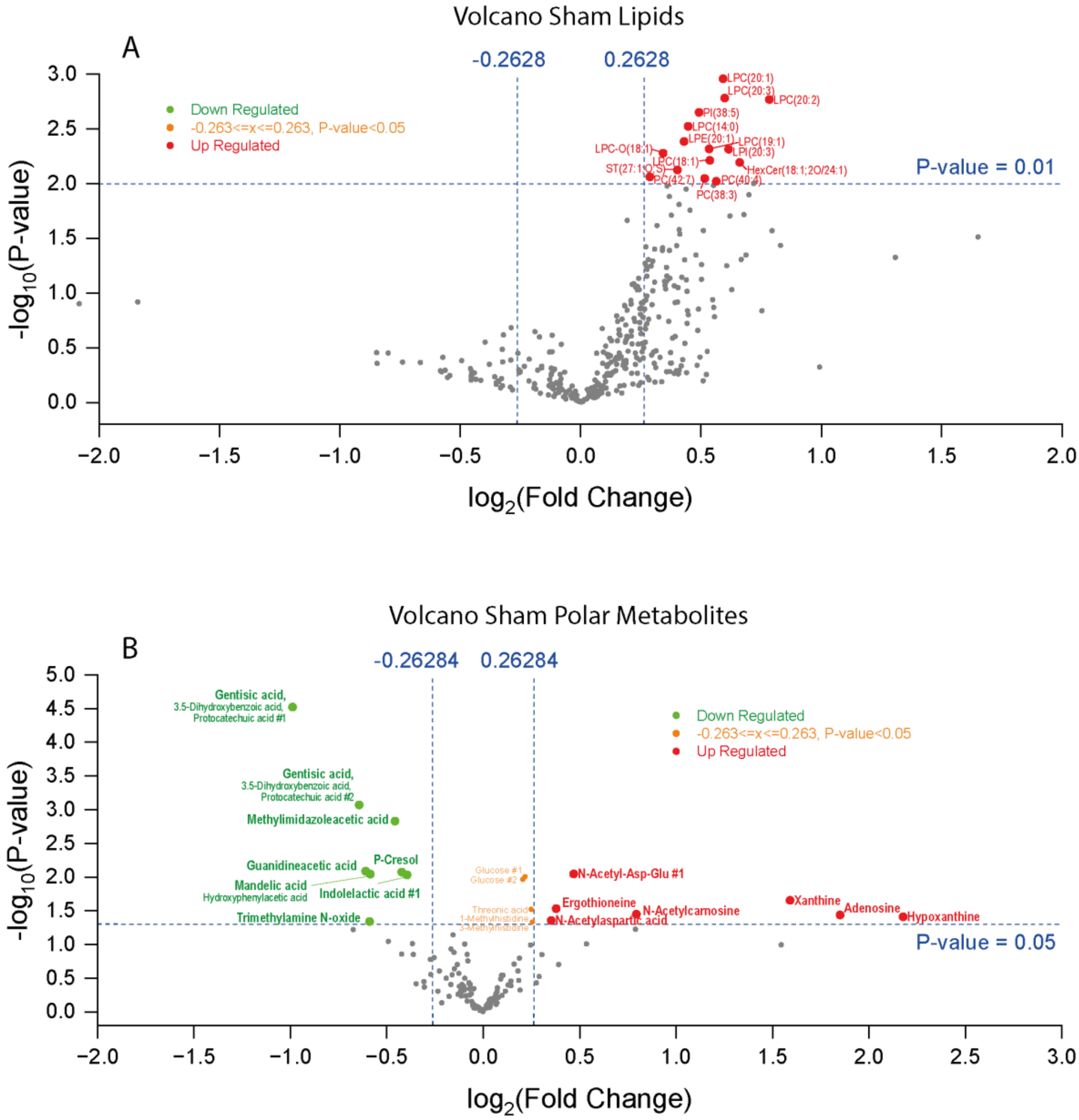

Lipidomic and metabolomic analyses of plasma samples 7 days after sham surgery in animals treated with vehicle or VPA (3 dpi 400 mg/kg VPA i.p.). **A:** Volcano plot of lipids of sham-vehicle versus sham-VPA mice. **B:** Volcano plot of polar metabolites of sham-vehicle versus sham-VPA mice. Lipids or metabolites increased in the VPA group are on the right side of the x-axis.

Supplementary Figure S5

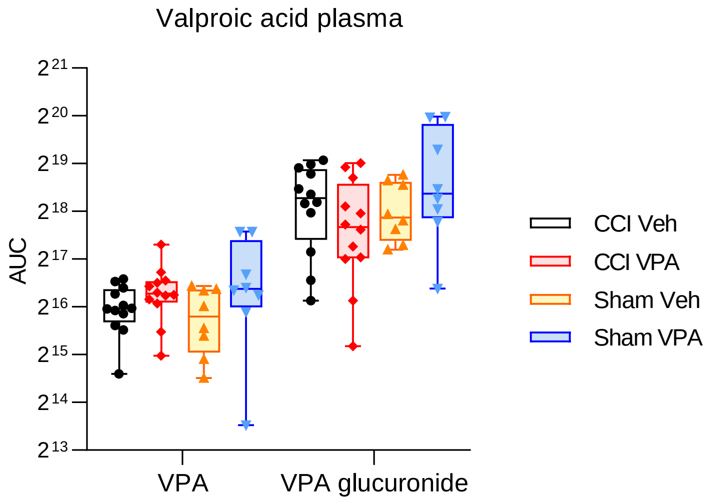

Valproic acid in plasma 7 dpi as assessed by UHPLC-Orbitrap-MS. The treatment period was day 1-3 after CCI/sham (400 mg/kg i.p. per day).

Supplementary Figure S6

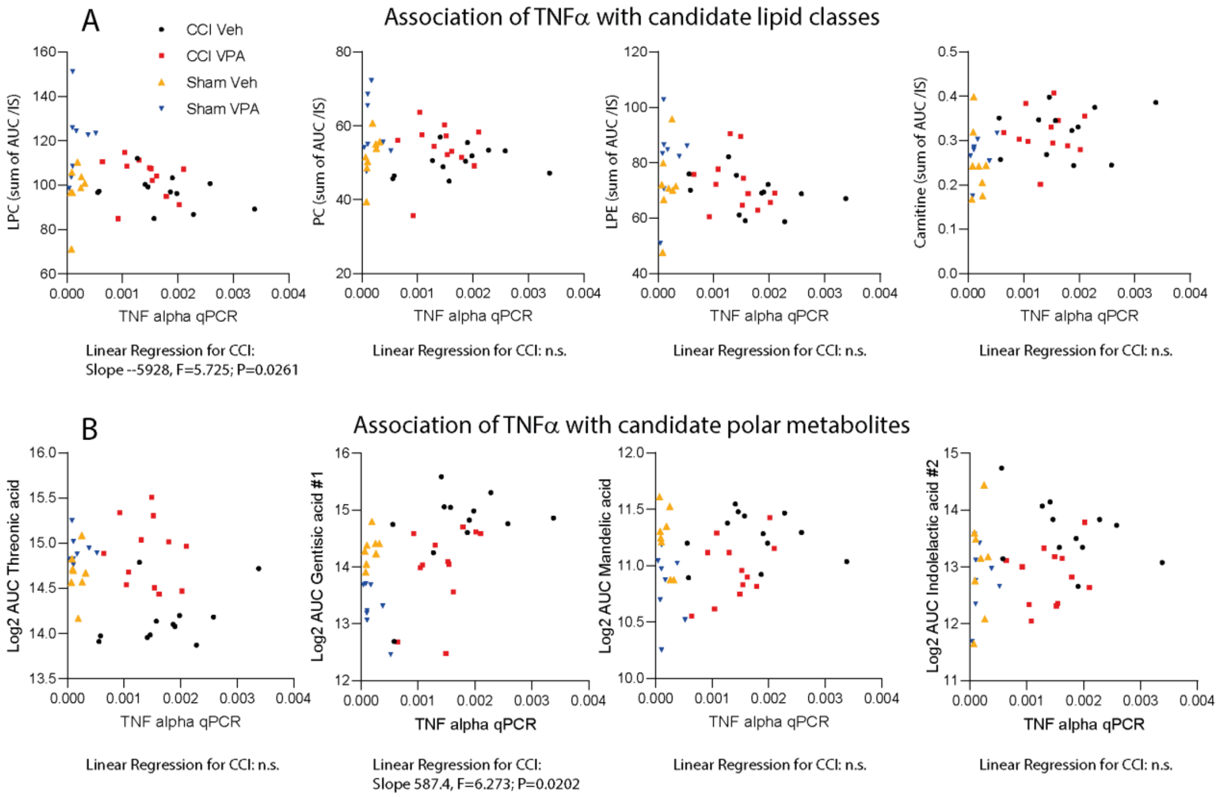

Association of lipid classes versus TNFα. To obtain a summary value of a lipid class, individual AUC/IS values of individual lipid species with different chain length and saturation of the respective class were summed. Associations with IL-1β are shown in Figure 8.

Sample sizes: controlled cortical impact (CCI) vehicle n=12, CCI VPA n=12, sham vehicle n=8, sham VPA n=8. Abbreviations of lipids: CAR, carnitines; CER, ceramides; LPC, lysophosphatidylcholine; PC, phosphatidylcholine; LPE, lysophosphatidylethanolamine; SE, steryl ester; SM, sphingomyelins; ST, sterols

Supplementary Figure S7

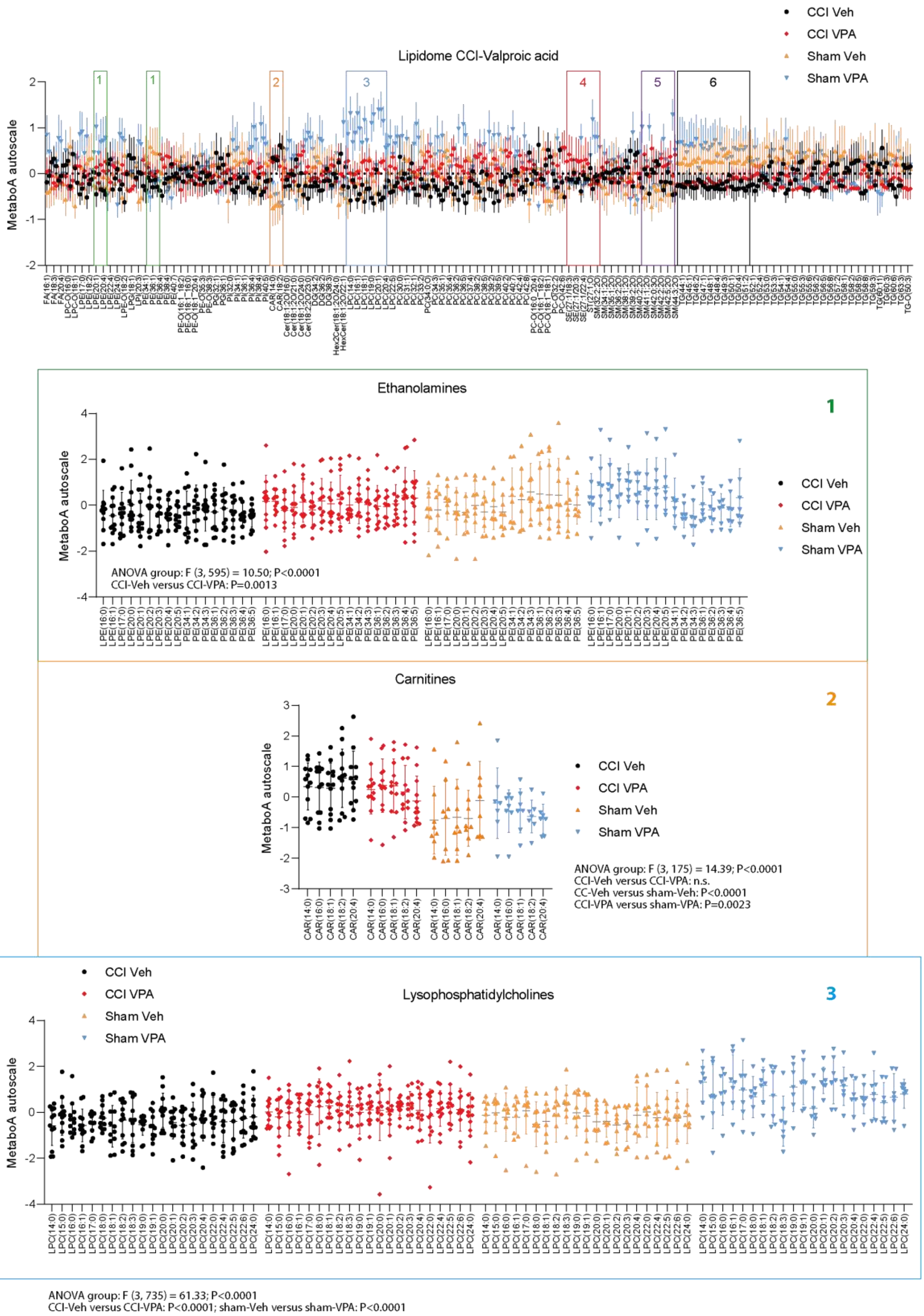

Overview of lipid species and magnified regions of interest of the lipidome, which are highlighted as numbered rectangles. Mice were treated with VPA or vehicle from 1-3 dpi (400 mg/kg i.p. per day)

and plasma samples were obtained 7 dpi. Scatters show individual mice. Sample sizes: controlled cortical impact (CCI) vehicle n=12, CCI VPA n=12, sham vehicle n=8, sham VPA n=7 (1 mouse with very high lipids were excluded). Data were autoscaled to have a mean of 0 and a variance of 1.

Rectangles-1: Lysophosphatidylethanolamides (LPE) that are increased particularly in sham-VPA treated mice and corresponding phosphatidylethanolamides (PE) which were decreased.

Rectangles-2: Long chain carnitines (CAR) which were equally increased in both CCI groups as compared with sham treated mice.

Rectangles-3: Lysophosphatidylcholines (LPC) which were strongly increased in sham-VPA mice and somewhat less increased in CCI-VPA mice.

Supplementary Figure S8

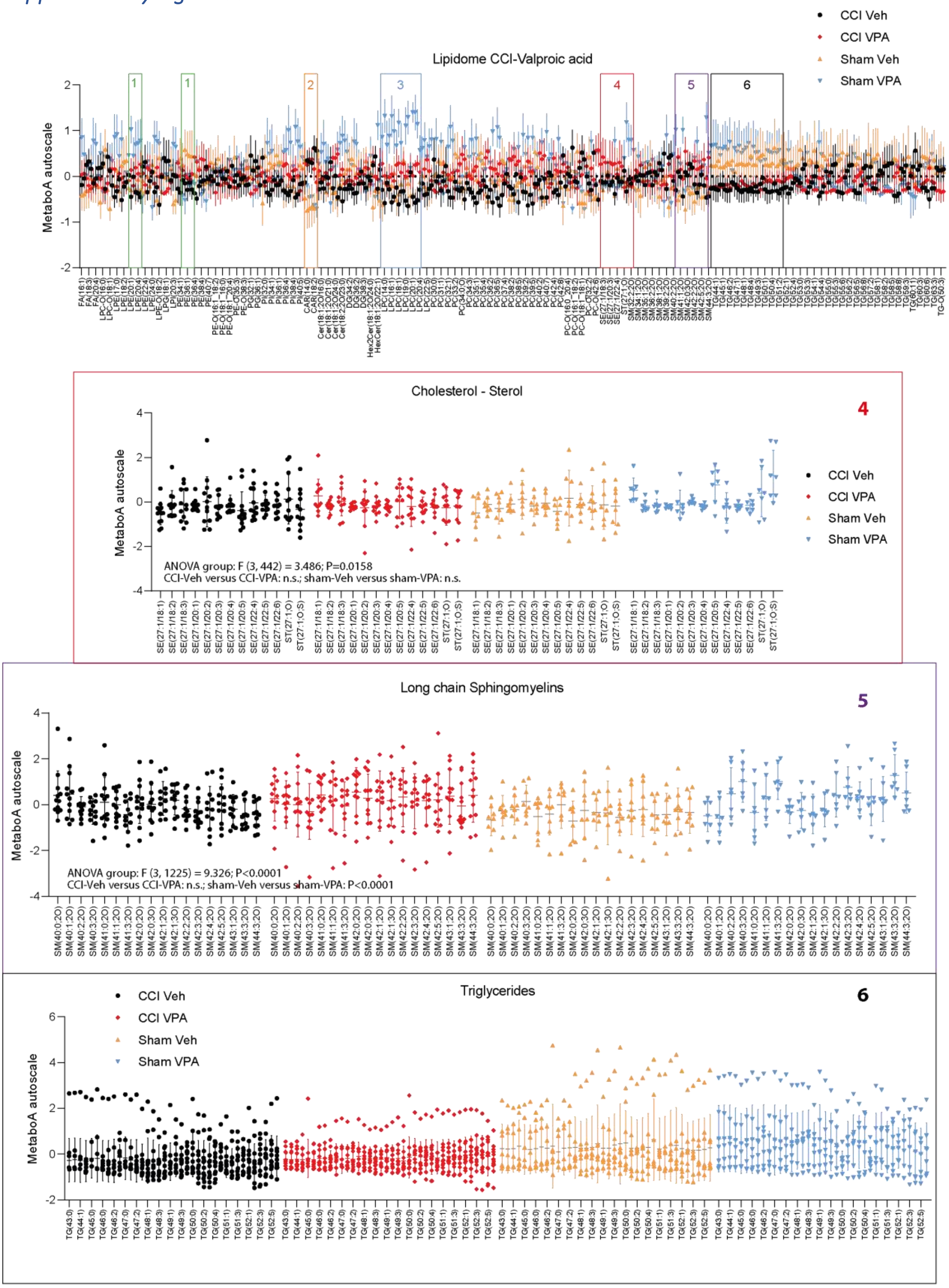

ANOVA group: F (3, 1330) = 41.35; P<0.0001
CCI-Veh versus sham-Veh: P<0.0001; CCI-VPA versus sham-VPA: P<0.0001; CCI-Veh versus CCI-VPA: n.s.; sham-Veh versus sham-VPA: n.s

Continuation of Suppl. Figure 7. The upper panel shows the overview.

Rectangle 4: Cholesterolester (SE 27:1/…) and sterols (ST). Mostly, levels were similar in all groups except for example SE 27:1/18:1 which is highly abundant and was increased in VPA treated mice both CCI and sham.
Rectangle 5: Long chain sphingomyelins (SM) which were increased in both VPA groups as compared to vehicle treated groups. There was no difference in shorter SM (not shown).
Rectangle 6: Short chain triglycerides (triacylglycerols, TG) which were higher in both sham groups as compared to the CCI treated mice suggesting higher de novo TG synthesis in sham animals. There was no difference in long to very long chain TG (C-atoms 54-60).
